# Two cases of multiple-drug-resistant adult-onset Still's disease treated successfully with tocilizumab - the relationship between interleukin 6 and 18

**DOI:** 10.1186/ar3663

**Published:** 2012-02-09

**Authors:** Kojiro Sato, Akinori Yamamoto, Yoshihiro Yoshida, Toshihide Mimura

**Affiliations:** 1 Department of Rheumatology and Applied Immunology, Saitama Medical University, Saitama 350-0495, Japan

## Background

Adult-onset Still's disease (AOSD) is an inflammatory disease of unknown cause characterized by a high spiking fever, arthritis and evanescent rash. The mainstay of treatment is glucocorticoids with or without immunosuppressants. Recently, biologics such as anti-tumor necrosis factor (TNF) antibodies have also been tried in certain refractory cases.

## Results

**Figure 1:**
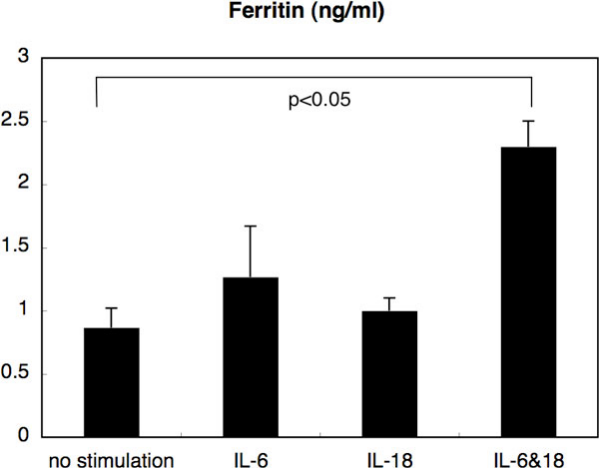
The level of ferritin in the supernatant of monocytes cultured with or without the presence of IL-6 and/or IL-18 (10 ng/mL each)

We have had two cases of AOSD which were treated successfully with anti-interleukin (IL-) 6-receptor antibody, tocilizumab (TOC). (Case 1) A 36-year-old woman who was diagnosed 8 years previously, and had been treated with various DMARDs plus etanercept (ETA) or adalimumab, presented with a high spiky fever and elevated liver enzymes. After excluding infection, she was treated with TOC. (Case 2) A 26-year-old man with new-onset AOSD, which was shown to be resistant to multiple immunosuppressants including infliximab and ETA, was treated with TOC starting 7 months after the diagnosis. In both cases, serum IL-18 was extremely high, and TOC promptly improved clinical symptoms and liver function. The high level of serum ferritin also became normalized. Interestingly, especially in case 2, the level of IL-18 remained high after the administration of TOC, suggesting that IL-18 is located either upstream of, or at the same level as, IL-6 in the pathogenesis of AOSD. Next, we cultured human monocytes derived from healthy controls with or without the presence of IL-6 and/or IL-18 *in vitro*. The level of ferritin in the supernatant was significantly increased only when both IL-6 and IL-18 were added, indicating that IL-6 and IL-18 have a synergistic effect on the production of ferritin (Figure 1).

## Conclusion

TOC can be a first-line biologic applicable against multiple-drug-resistant AOSD. If an IL-18 blocker is developed, however, it may be even more beneficial in that it may block the cascade of inflammation at a point further upstream.

